# Revision Surgery in Total Joint Replacement Is Cost-Intensive

**DOI:** 10.1155/2018/8987104

**Published:** 2018-09-25

**Authors:** Markus Weber, Tobias Renkawitz, Florian Voellner, Benjamin Craiovan, Felix Greimel, Michael Worlicek, Joachim Grifka, Achim Benditz

**Affiliations:** ^1^Department of Orthopaedic Surgery, Regensburg University Medical Center, Asklepios Klinikum Bad Abbach, Kaiser-Karl V.-Allee 3, 93077 Bad Abbach, Germany; ^2^Department of Trauma Surgery, Regensburg University Medical Center, Franz-Josef-Strauß-Allee 11, 93053 Regensburg, Germany

## Abstract

Revisions after total joint replacement increase constantly. In the current study, we analyzed clinical outcome, complication rates, and cost-effectiveness of revision arthroplasty. In a retrospective analysis of 162 revision hip and knee arthroplasties from our institutional joint registry responder rate, patient-reported outcome measures (EQ-5D, WOMAC), complication rates, and patient-individual charges in relation to reimbursement were compared with a matched control group of primary total joint replacements. Positive responder rate one year postoperatively was lower for revision arthroplasties with 72.9% than for primary arthroplasties with 90.1% (OR=0.30, 95%CI=0.18–0.59, p=0.001). Correspondingly, improvement in patient-reported outcome measures one year after surgery was lower in revision than in primary joint arthroplasty with EQ-5D 0.19±0.25 to 0.30±0.24 (p<0.001) and WOMAC 24.3±30.3 to 41.2±21.3 (p<0.001). Infection rate was higher in revision (6.8%) compared to primary replacements (0%, p=0.001). Mean charges in revision arthroplasty were 76.0% higher than in matched primary joint replacements (7110.8±2249.4$ to 4041.1±975.7$, p<0.001), whereas reimbursement was only 23.6% higher (9243.3±2258.4$ in revision and 7477.9±703.1$ in primary arthroplasty, p<0.001). Revision arthroplasty is associated with lower outcome and higher infection rate compared to primary replacements. The high financial expense of revision arthroplasty is only partly covered by a higher reimbursement.

## 1. Introduction

In orthopaedic surgery total hip and knee replacements are one of the most successful and frequently performed procedures [1]. They represent a curative treatment option of advanced hip and knee osteoarthritis with the capacity to substantially improve pain, function, and quality of life [2]. Despite continuous improvement in surgical technique and implant design the number of revision arthroplasties is still expected to grow [3]. By the year 2013 total hip and total knee arthroplasty is projected to increase by 137% and 601%, respectively, in the United States [4]. The most common reasons for revision total joint arthroplasty reported in literature are instability, aseptic loosening, and infection [5–7].

Revision arthroplasty is a complex and challenging procedure. The associated resource consumption substantially differs from primary total joint replacements [8]. From a socioeconomic point of view the high numbers of revision arthroplasty represent a financial burden [3]. As demonstrated in previous studies, the average hospital cost for revision total hip arthroplasty has more than tripled within a period of ten years [5, 6, 8]. In literature the percentage of patients undergoing revision arthroplasty in relation to primary total joint arthroplasties is described as the revision arthroplasty burden [4]. Despite all technical progress and surgical efforts this revision burden has not decreased over the past decades [9].

Independent of financial aspects orthopaedic surgeons aim for the best operative treatment in patients undergoing revision arthroplasty. However, a considerable number of patients still complain about residual pain and restricted function [10]. Furthermore, revision arthroplasty is supposed to be associated with higher postoperative complication rates and longer hospital stay [11, 12]. However, advances in joint arthroplasty over the last two decades might have reduced complication rates.

In the current retrospective analysis of 162 revision total hip and knee replacements and corresponding 162 sex, age, and ASA (American Society of Anaesthesiologists) class matched primary total joint replacements we aimed to investigate responder rate, early clinical outcome, complication rates, and economic parameters such as operative time and length of hospital stay, and patient-individual charges in relation to reimbursement at a high volume arthroplasty centre.

## 2. Patients and Methods

A retrospective analysis of revision hip and knee replacements and a matched control group of corresponding primary replacements from our institutional joint registry was performed [14]. The local Ethics Commission waived approval due to the retrospective study design. A power calculation was performed for the investigation of the primary endpoint positive responder rate after revision total hip and knee arthroplasty. The corresponding hypothesis was tested on a 5% significance level. Derived from a previous study [10] and our own clinical data the expected difference in responder rates was set to 10%. Based on these considerations, a sample size of 151 in each group achieved a power of 80% using two-sample chi-square test (nQuery Advisor 7.0, Statistical Solutions Ltd., Cork, Ireland). From the database 162 patients undergoing all component revision after total hip and knee replacement with complete postoperative outcome measures were chosen. Patients with liner exchange, soft tissue revision, and incomplete data files were excluded. This group was matched with a control group of primary total hip and knee replacements according to sex, age, and ASA class. A total of 162 matched pairs were available for final analysis. All operations were performed between January 2012 and December 2016 at Department of Orthopaedic Surgery at Regensburg University Medical Center, Germany. Available data from the institutional joint registry included patient age, sex, ASA class, operative procedure, operative time, length of hospital stay, infection rate, and pre- and one-year postoperative Western Ontario and McMaster Universities Arthritis Index (WOMAC) [15] and Euro-Quol 5D-5L (EQ-5D) [16]. Using the anonymized case numbers of the registry numbers patient-individual charges such as implant charges, perioperative charges, and charges for hospital stay as well as overall reimbursement were available from our financial controlling department. The WOMAC is an international widely used score to evaluate outcome after total joint replacement representing a multidimensional measure of pain, stiffness, and physical functional disability [17]. This measurement of outcomes by health-related quality of life questionnaire has especially been developed for patients with osteoarthritis and has been approved in several longitudinal studies with patients undergoing total joint replacement [18–20]. The EQ-5D is a widely used and tested descriptive instrument for evaluating health. It defines health based on five dimensions: Mobility, Self-Care, Usual Activities, Pain/Discomfort, and Anxiety/Depression. To improve the instrument's sensitivity to small and medium health changes and to reduce ceiling effects the number of levels of severity in each dimension was expanded in 2005 to a five-level descriptive system increasing reliability and sensitivity of EQ-5D [16].

Altogether 94 matched pairs were available for revision hip arthroplasty and 68 for revision knee arthroplasty, respectively. Anthropometric characteristics of the study group are shown in Table 1. Revision total hip arthroplasty in all patients was performed in the supine decubitus position using a lateral Hardinge approach. In the control group, a minimally invasive single-incision anterolateral approach to the hip was used in terms of an intermuscular and interneural tissue plane between the tensor muscle and the gluteus medius muscle [21]. Data of the components implanted for revision were not available in our data base. For primary cementless total hip arthroplasty press-fit acetabular components and cement-free hydroxyapatite-coated stems of one single manufacturer (Pinnacle®cup, Corail®stem or Trilock®stem, DePuy, Warsaw, IN, USA) were used. Both primary and revision total knee arthroplasty in all patients were performed through a standard medial parapatellar approach including a tourniquet. Data of the components implanted for revision were not available in our data base. For primary knees cemented components of one single manufacturer (PFC Sigma®, DePuy, Warsaw, IN, USA) were used in all total knee replacements. No patella resurfacing was performed.

For dichotomizing responders and nonresponders within the first year after surgery, the Outcome Measures in Rheumatology and Osteoarthritis Research Society International consensus responder criteria (OMERACT-OARSI) [13, 15] were used as previously described [22]. These criteria assess responder status based on relative change in Index (WOMAC) scores in relation to benchmarks determined by expert consensus and statistical analyses. OMERACT-OARSI criteria were chosen since they do not depend on patient characteristics of the cohort and thus reducing any potential selection bias due to the retrospective design of the study [23]. The OMERACT-OARSI criteria to assess responders after total joint replacement include improvement in pain or function of at least 50% and absolute change of at least 20 points. Alternatively, responders are also defined by fulfilment of two of the following criteria: improvement in pain of at least 20% and absolute change of at least 10 points, improvement in function of at least 20% and absolute change of at least 10 points, or global improvement of at least 20% with absolute change of at least 10 points [13].

For statistical analysis, continuous data are presented as mean (standard deviation). Group comparisons were performed by two-sided t-tests. Absolute and relative frequencies were given for categorical data and compared between groups by chi-square tests. The primary hypothesis in the study was tested on 5% significance level. For all secondary hypotheses, significance levels were adjusted according to Bonferroni [24]. Odds ratio (OR) and 95% confidence interval (95% CI) were estimated by logistic regression. IBM SPSS Statistics 22 (SPSS Inc., Chicago, IL, USA) was used for analysis.

## 3. Results

**Figure 1:**
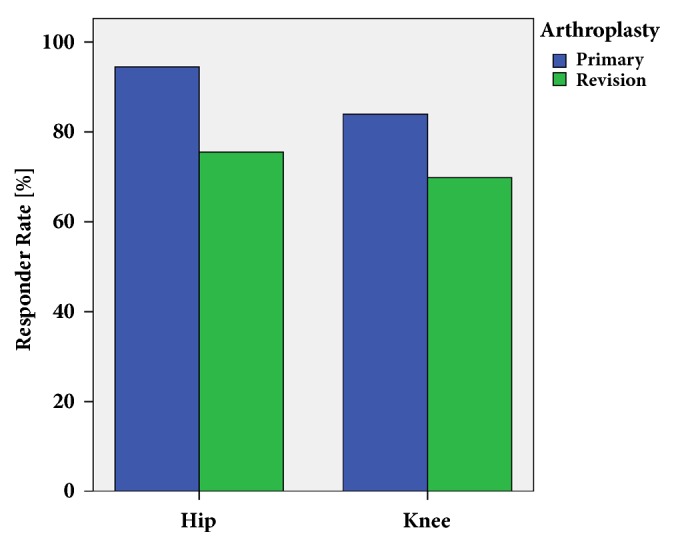
Responder rate as defined by the OMERACT-OARSI criteria [13] one year after revision arthroplasty of the hip and knee compared to matched primary hip and knee replacements.

**Figure 2:**
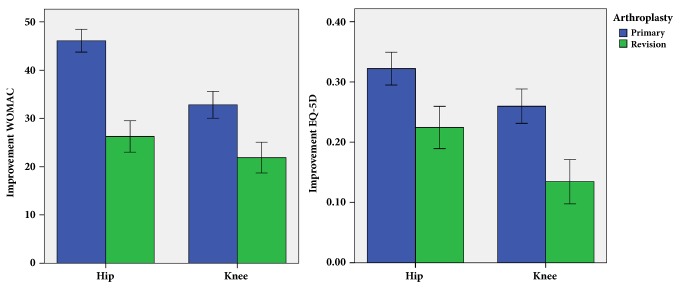
Improvement of patient-reported outcome measures (WOMAC, EQ-5D) within the first year after revision total joint arthroplasty.

The positive responder rate as defined by the OMERACT-OARSI criteria [13] within the first year after surgery was lower for total revision total hip and knee arthroplasty with 72.9% (118/162) compared to matched control primary total hip and knee replacements with 90.1% (146/162, OR = 0.30, 95% CI = 0.18 – 0.59, p=0.001, Figure 1). Researching into patient-reported outcome measures one year postoperatively WOMAC scores showed a lower improvement for revision arthroplasty (24.3 ± 30.3) compared to primary total joint arthroplasty (41.2 ± 21.3, p<0.001). Accordingly increase of EQ-5D values one year after surgery was lower in the revision group (0.19 ± 0.25) than in the matched control group of primary total joint replacements (0.30 ± 0.24, p<0.001, Figure 2). Analyzing outcome measures subscores, again one-year results were lower in patients undergoing revision than those with primary total joint replacement (Table 2).

Researching into adverse events, we found a higher infection rate in revision (6.8%, 11/162) compared to primary arthroplasty (0.0%, 0/162, p=0.001), whereas no differences were observed regarding intraoperative fractures, thrombosis, or neurologic deficits (Table 3).

**Figure 3:**
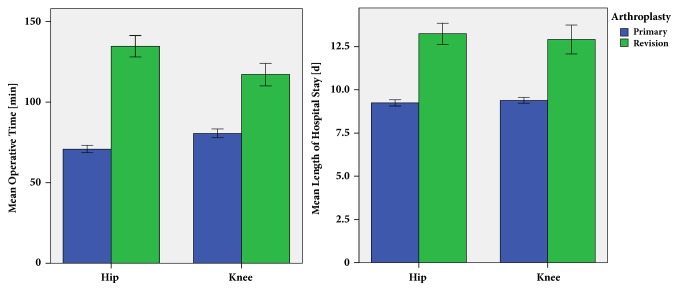
Mean operative time and length of hospital stay of revision total hip and knee arthroplasty compared to control group of primary total joint replacements.

**Figure 4:**
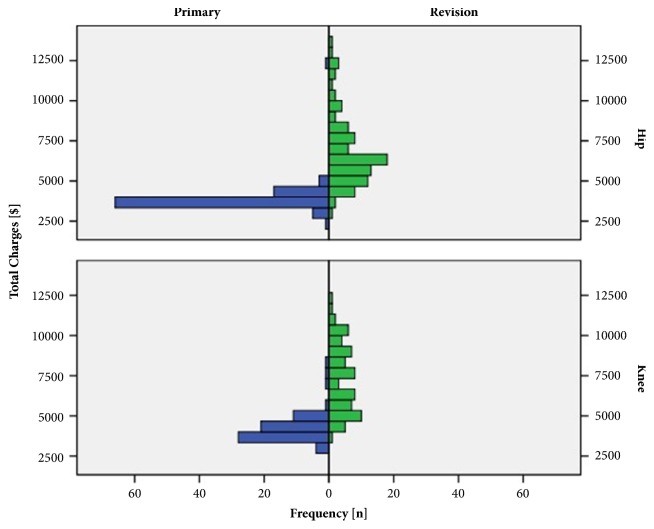
Distribution of charges for revision total hip and knee arthroplasty compared with matched primary total joint replacements.

Mean operative time was 52 minutes longer for revision total joint replacement compared to matched primary total hip and knee replacements (127.0 ± 61.3 min versus 74.9 ± 22.6 min, p<0.001). Similarly, patients undergoing joint revision had a longer hospital stay of 4 days compared to the control group (13.1 ± 6.3 d versus 9.3 ± 1.6 d, p<0.001, Figure 3). Researching into socioeconomic aspects, revision arthroplasty resulted in a higher financial expense of 76.0% compared with matched primary joint replacements (7110.8 ± 2249.4$ to 4041.1±975.7$, p<0.001, Figure 4). The increased charges in revision arthroplasty were due to higher implant costs, perioperative costs, and costs of hospital stay (p<0.001). The higher reimbursement of 23.6% (9243.3±2258.4$ in revision to 7477.9±703.1$ in primary arthroplasty, p<0.001) did only partly cover the elevated costs for revision joint replacements (Table 4).

## 4. Discussion

Primary total hip and knee arthroplasty is a frequently performed and successful procedure in orthopaedic surgery [25]. Correspondingly, the number of revision arthroplasties increases and is associated with considerable financial expense [3]. In the current retrospective study, we aimed to compare (1) responder and early clinical outcome within the first year after total joint replacement, (2) complication rate, and (3) patient-individual charges in relation to reimbursement between revision and primary total hip and knee arthroplasty. We found a lower responder rate and lower clinical outcome for revision arthroplasty than for matched primary total joint replacements. Infection rate was higher in the revision group. In general revision arthroplasty required 52 minutes longer operative time and a prolonged hospital stay of 4 days compared to matched primary arthroplasties. In addition to higher implant costs this resulted in higher charges of 76.0% compared to primary hip and knee replacements.

There are several limitations of this study. First, the study design is a retrospective analysis. Therefore, the results are susceptible to potential bias. We tried to reduce this and matched the cohort in terms of age, ASA, and sex. To further minimize potential bias we chose patient characteristics independent dichotomization for responders. Using noncohort dependent benchmarks should maximize generalizability. Second, the current study is restricted to the information provided by the institutional joint registry. Other parameters such as the patient's psychological or social status might have an impact on the patient specific outcome and improve prediction of outcome. Third, for the current analysis only short-term outcome data for the first 12 months were available. It would have been of interest to include long-term outcome and failure rates. Fourth, we were not able to differentiate between the reasons and types of revision surgery. All operations were all component revisions. However, this included easier and extraordinary challenging procedures. A strength of the study is the fact that all data refer to one single university medical centre reflecting a specific operative workflow for total hip and knee replacement as well as an identical postoperative treatment protocol for all patients. This contributes to minimizing confounding factors.

In answer to the first question of the study, we found an excellent responder rate within the first year as defined by the OMERACT-OARSI criteria [13] after primary total hip and knee replacement with 90.1%. This is in line with other studies underlining the benefit from total joint replacement [1, 2]. In contrast, responder rate in all component revision surgery after hip and knee arthroplasty was significantly lower with 72.9%. Similarly, residual pain after revision arthroplasty has been described in literature [10]. According to the lower responder rate in all component revision total hip and knee arthroplasty, patient-reported outcome measures as assessed by WOMAC and EQ-5D differed between revision and primary total joint replacement one year after surgery. However, the outcome data after revision were on a higher level compared to previous data in literature [26]. This demonstrates that in modern revision arthroplasty still good outcome is achievable. Overall outcome measures for primary total joint replacement in our study were similar to previous published early results after total joint replacement of the hip and knee, respectively [27–31]. Furthermore, our data are supported by a previous study showing poorer functional outcome for knee replacements compared to hip replacements [32].

In addition we analyzed complication rates after joint replacement since the risk of severe adverse events such as infection or fracture has to be considered and balanced with the potential benefit of revision arthroplasty. However, except for infection complication rates were comparably low for both revision and primary arthroplasty emphasizing revision arthroplasty represents a safe procedure in orthopaedic surgery. The observed results are in accordance with literature [27, 33, 34]. Regarding infection rate there was markedly higher number of infections after revision compared to primary arthroplasty. One reason for this higher rate might be due to the fact that revisions due to infection were included in the revision cohort. In relation to previous results in literature, the infection rate was still within the lower range [35]. No differences between the revision and primary arthroplasty group were observed regarding thrombosis and neurological deficits with the numbers available.

Researching into economic relevant data, mean operative time was 52 minutes longer for revision arthroplasties compared to matched primary arthroplasties. The increase in operative time was more apparent in hip than in knee revisions compared to matched primary total joint replacements. Compared to data from revision arthroplasty two decades ago operative times for revision arthroplasty have decreased by 50 percent nowadays [12]. The observed operative times for both primary hip and knee replacements were comparable to modern literature [27]. From an economic point of view, a prolonged operative time means higher financial expense. In addition, mean hospital stay was 4 days longer in our study cohort for revision arthroplasty compared to matched primary cases. A prolonged hospital stay for revision arthroplasty has been previously reported in literature [36]. This further adds costs to the public health care sector as well as higher implant costs resulting in higher procedural charges [3]. In our study cohort charges for revision arthroplasty were 76.0% higher compared to primary total joint replacements. In contrast, reimbursement was 23.6% higher in revision compared to primary arthroplasty and thus did only partly cover the high charges. In addition charges for revision arthroplasty are still rising. In previous studies a threefold increase of overall costs for revision hip arthroplasty over the last decade has been calculated [6, 8]. Mean annual economic revision burdens of 27% have been reported in literature for revision total hip and knee arthroplasties [3]. By 2013 the demand for revision hip and knee arthroplasty is expected to substantially grow [4]. Therefore, revision arthroplasty of the hip and knee represents a severe challenge for public health care systems. On the other hand the increase in outcome after revision might lead to a decrease of costs in the period after surgery from the perspective of both patient [19] and public health care [37].

## 5. Conclusions

In conclusion, both hip and knee revision arthroplasty enable patients to regain good function and outcome. Still, patients experience lower outcome compared to primary total joint replacement. Despite higher infection rates revision arthroplasty is a safe procedure with tolerable complication rates. However, revision total hip and knee arthroplasty is cost-intensive and thus a challenge for public health care.

## Figures and Tables

**Table 1:** Anthropometric characteristics of the study group*∗*.

	Hip Revision	Hip Primary	Knee Revision	Knee Primary
Number of patients	94	94	68	68

Age (years)	66.9 ± 14.0	66.9 ± 14.0	67.9 ± 9.2	67.9 ± 9.2

Gender (men/women)	41/53	41/53	26/42	26/42

ASA-Class 1	12 (12.8%)	12 (12.8%)	3 (4.4%)	3 (4.4%)

ASA-Class 2	40 (42.6%)	40 (42.6%)	30 (44.1%)	30 (44.1%)

ASA-Class 3	42 (44.7%)	42 (44.7%)	35 (51.5%)	35 (51.5%)

*∗*For categorical data values are given as relative and absolute frequencies and for quantitative data values are given as mean (standard deviation); ASA = American Society of Anaesthesiologists.

**Table 2:** Western Ontario and McMaster Universities Arthritis Index (WOMAC) and Euro-Qol 5D-5L (EQ-5D) for revision and primary total hip and knee arthroplasty preoperative and 1 year after surgery*∗*.

**Joint replacement**		EQ-5D preop	EQ-5D postop	WOMAC preop	WOMAC postop	Pain preop	Pain postop	Stiffness preop	Stiffness postop	Function preop	Function postop
**Hip** **Revision**	mean	0.50	0.71	41.17	68.07	40.00	76.00	44.91	69.68	40.84	65.00
	SD	0.25	0.27	20.43	22.87	23.47	22.72	28.48	22.77	21.07	24.64

**Hip** **Primary**	mean	0.54	0.85	38.06	84.00	36.22	86.28	40.63	81.85	37.83	82.68
	SD	0.24	0.20	17.35	18.36	19.98	17.40	21.63	21.53	18.57	20.06

p-value		0.45	<0.001	0.37	<0.001	0.31	0.001	0.31	<0.001	0.40	<0.001

**Knee** **Revision**	mean	0.59	0.71	43.41	63.18	40.81	64.93	44.77	61.21	44.32	63.24
	SD	0.20	0.23	14.84	23.62	18.29	25.52	23.02	24.73	15.82	23.67

**Knee** **Primary**	mean	0.51	0.78	37.76	70.57	34.14	74.31	36.29	65.00	38.85	69.81
	SD	0.21	0.19	12.45	19.12	14.32	18.20	21.32	20.52	13.61	19.62

p-value		0.10	0.08	0.05	0.06	0.04	0.02	0.06	0.34	0.07	<0.10

*∗*For quantitative data values are given as mean (SD = standard deviation). preop = preoperative. postop = postoperative.

**Table 3:** Complication rates for revision and primary arthroplasty of the hip and knee*∗*.

**Total Joint Replacement**	Hip Revision	Hip Primary	Knee Revision	Knee Primary
Intraoperative fractures	0.0% (0/94)	1.1 % (1/94)	0.0% (0/68)	0.0% (0/68)

Thrombosis	0.0% (0/94)	0.0% (0/94)	0.0% (0/68)	0.0% (0/68)

Neurological deficits	1.1% (1/94)	0.0% (0/94)	0.0% (0/68)	1.5% (1/68)

Joint infection	5.3% (5/94)	0.0% (0/94)	8.8% (6/68)	0.0% (0/68)

*∗* For categorical data values are given as relative and absolute frequencies.

**Table 4:** Financial expense of revision arthroplasty compared to primary joint replacement*∗*.

**Cost Analysis**	Hip Revision	Hip Primary	Knee Revision	Knee Primary
Implant	2240.5 (1163.7)	978.2 (445.9)	3052.3 (1178.8)	1155.8 (707.9)

Perioperative	2018.8 (947.6)	1062.1 (327.0)	1755.2 (863.8)	1207.9 (337.7)

Hospital stay	2649.0 (1183.9)	1849.4 (364.6)	2582.8 (1374.6)	1878.1 (270.3)

Combined	6908.3 (2312.2)	3889.7 (994.9)	7390.4 (2148.2)	4241.8 (918.3)

DRG-Income	8920.6 (2084.8)	7225.7 (643.1)	9689.3 (2423.8)	7826.6 (633.4)

Difference	2012.3 (2576.5)	3336.0 (667.0)	2299.0 (2227.9)	3584.7 (689.2)

p-value	p<0.001 for all variables		p<0.001 for all variables	

*∗* For quantitative data values are given as mean (SD = standard deviation).

## Data Availability

The data used to support the findings of this study are available from the corresponding author upon request.
